# Can resident registration expiration statistics due to death be used for near-real-time mortality tracking? A validation study using 2023 data from Korea

**DOI:** 10.4178/epih.e2025042

**Published:** 2025-08-03

**Authors:** Jin-Hwan Kim

**Affiliations:** Institute for Health and Environment, Seoul National University, Seoul, Korea

**Keywords:** Validation study, Population health, Mortality, Republic of Korea

## Abstract

**OBJECTIVES:**

Real-time mortality tracking is essential for public health surveillance, especially during emergencies such as the coronavirus disease 2019 (COVID-19) pandemic. In Korea, delayed availability of vital statistics (VS) data has hindered timely mortality monitoring. This study evaluates whether National Administrative Data (NAD) on resident registration expiration due to death, provided by the Ministry of Interior and Safety, could serve as a reliable alternative for near real-time mortality surveillance.

**METHODS:**

We compared mortality counts between VS and NAD for 2023 at multiple geographic levels (county, province, and nation) and across demographic strata (sex and age groups). The analysis was conducted in 3 stages: comparing overall mortality counts, analyzing county-level distributions, and assessing equivalence through correlation analyses, scatter plots, and density plots.

**RESULTS:**

NAD showed strong agreement with VS at the national level, reporting only 0.4% more deaths overall (0.2% for male, 0.6% for female). Notable differences were observed in early childhood mortality, with NAD showing 16.8% fewer deaths for neonates (age 0) and 14.8% more for ages 1-4, as well as in monthly variations (5-9%). Correlation analyses indicated extremely high consistency between the 2 data sources across all geographic levels (correlation coefficients ≥0.999), especially at the national and provincial levels.

**CONCLUSIONS:**

NAD provides a reliable alternative to VS for real-time mortality surveillance in Korea, providing comparable accuracy with much-improved timeliness. Although some variations are present in specific age groups and monthly trends, these can be addressed through appropriate analytical strategies. The recent availability of sex-specific and age-specific data in NAD since 2023 establishes it as a valuable infrastructure for mortality surveillance.

## GRAPHICAL ABSTRACT

[Fig f3-epih-47-e2025042]

## Key Message

• National Administrative Data (NAD) demonstrates high concordance with vital statistics (VS), making it applicable for establishing rapid mortality surveillance systems.

• With the inclusion of sex- and age-specific information since 2023, NAD has evolved into a critical mortality surveillance infrastructure for Korea’s public health emergency response.

## INTRODUCTION

The coronavirus disease 2019 (COVID-19) pandemic has underscored the critical importance of rapidly tracking and monitoring changes in mortality rates, leading countries worldwide to calculate and monitor excess mortality. International initiatives such as the International Mortality Database [1], the EuroMOMO network [2], and the Global Burden of Disease Excess Mortality Collaborator [3] have contributed to consolidating death data from different countries to provide a comprehensive global perspective.

In Korea, despite these global efforts, several studies have attempted to estimate excess mortality during the COVID-19 pandemic but have largely failed to monitor mortality changes in real or near-real time [4-9]. This limitation primarily stems from the reliance on vital statistics (VS) from Statistics Korea as the sole publicly available source for mortality tracking. VS data becomes available only after October of the following year, resulting in a lag of up to 20 months between the occurrence of death and data release (e.g., mortality data from January 2023 is accessible in October 2024).

Although the National Health Insurance Service (NHIS) is known to acquire mortality data relatively quickly through administrative sources, and some excess mortality analyses [5,7] have utilized this data, real-time or near-real-time mortality tracking has not yet been achieved. This is evident from the timelines of relevant publications: analyses of 2020 mortality were published in September 2022, and studies examining early 2022 mortality did not appear until September 2023. As a result, during the pandemic, the COVID-19 webpage (https://kosis.kr/covid/covid_index.do) provided by Statistics Korea was the only means of gaining a rough, real-time overview of mortality rate changes.

Another potential data source for near-real-time mortality tracking is the “expiration due to death” data from the National Resident Registration Register, managed by the Ministry of Interior and Safety (MOIS). MOIS releases these data at the start of each month, and this source, hereafter referred to as National Administrative Data (NAD), could establish a robust infrastructure for near-real-time mortality monitoring. Notably, because MOIS oversees the resident registration population, this population can readily be used as the denominator in NAD, providing a significant advantage over NHIS data, which require calibration to the population covered by health insurance or Medical Aid programs.

However, during the 2020-2022 pandemic, NAD did not provide sex-specific and age-specific death counts, and these details only became available in 2023. The equivalence between NAD and VS is supported indirectly through separate studies validating both NAD and VS against NHIS data [10,11]. Nevertheless, since Statistics Korea requires more than a year for death verification—and with the aim of using NAD as a real-time alternative to VS for mortality surveillance—a direct comparison of these 2 sources is necessary.

The primary objective of this study was to determine whether NAD can serve as a reliable alternative to VS for tracking mortality changes in real or near-real time. We compared mortality counts between NAD and VS at varying levels of detail. The analysis was conducted in 3 stages. First, we compared overall mortality counts between NAD and VS for 2023. Second, we analyzed the distribution of county-level mortality counts across the 2 datasets. Third, we assessed the equivalence of mortality counts between NAD and VS using correlation analyses, scatter plots, and density plots.

## MATERIALS AND METHODS

### Data

This study analyzed death registration data from 2 sources in Korea for the year 2023: VS from Statistics Korea and resident registration expiration due to death (NAD) from the MOIS. From both sources, we extracted death counts stratified by sex and by 5-year age groups (0, 1-4, 5-9, 10-14, ..., ≥85 years) at the administrative district (*si/gun/gu*; city/county/district) level. The VS data were obtained from the Microdata Integrated Service of Statistics Korea (https://mdis.kostat.go.kr), while NAD data were collected from the resident registration expiration statistics in the Resident Registration Demographics database maintained by MOIS (https://jumin.mois.go.kr).

### Unit of analysis

The minimal unit of analysis in this study was the city/county/district (*si/gun/gu*) level. Equivalence between VS and NAD was further evaluated by aggregating data into larger administrative units—the provincial and national levels. Sejong City, which lacks county-level administration, was treated as a single county for this analysis. Jeju Province, which is composed only of administrative cities, was represented by its 2 constituent cities: Jeju City and Seogwipo City. In total, 229 counties were included in the analysis.

### Statistical analysis

The analysis consisted of 3 stages: (1) mortality counts between VS and NAD were compared at the national level, with stratification by sex, province, month, and age group; (2) correlations between VS and NAD were assessed using Lin’s concordance correlation coefficient [12] across various combinations of age group, sex, and regional level; (3) the distribution of county-level mortality counts between the 2 datasets was examined using scatter plots and density plots.

In each stage, data were stratified by sex (total, male, and female) and categorized using 2 age grouping methods: 5-year intervals (0, 1-4, 5-9, ..., 80-84, ≥85) and broader life cycle stages (0-19, 20-64, 65-74, 75-84, ≥85). Geographic analyses were performed at 3 administrative levels: county, provincial, and national.

### Ethics statements

The institutional review board granted an exemption for this study (IRB No. E2502/004-005).

## RESULTS

Table 1 compares mortality data from VS (Statistics Korea) and NAD (MOIS). Because NAD was considered as a potential substitute for VS, NAD values are expressed as percentages relative to VS (set at 100). The nationwide comparison revealed minimal differences between the 2 sources. NAD reported 1,409 more deaths than VS, corresponding to a 0.4% difference. By sex, NAD recorded 420 (0.2%) more deaths for male and 989 (0.6%) more for female compared to VS.

At the provincial level, notable discrepancies appeared in Gyeongbuk (higher in NAD) and Daegu (lower in NAD), likely due to the administrative transfer of Gunwi County from Gyeongbuk Province to Daegu Metropolitan City in July 2023. Excluding these regions, all other provinces showed NAD counts approximately 1% higher than VS. Monthly comparisons revealed slightly greater variation than regional differences. NAD reported 5-9% more deaths than VS in February, March, June, and October, while showing lower counts in adjacent months—April, July, September, and December. These monthly trends were consistent according to gender, with only minor differences.

Table 2 presents the age-specific and sex-specific mortality distributions from VS and NAD. The most significant differences were observed in early childhood mortality (ages 0-4). Among neonates (age 0), NAD reports 16.8% fewer deaths than VS, with a larger discrepancy in male (20.6% lower) than female (11.9% lower). However, this trend reversed for ages 1-4, where NAD reported 14.8% more deaths than VS, with a greater difference in female (20.3% higher) than in male (9.9% higher). These contrasting patterns may be attributable to differences in the timing of death registration between the 2 systems.

For children and adolescents (ages 5-19), moderate differences persisted, with NAD consistently reporting 2-6% higher mortality than VS. Among adults aged 20-64, the differences became minimal, generally remaining within 1%. This pattern of small differences continued in the older population (65 years and older), with variations typically less than 1%. Two notable exceptions were the ≥85 age group, where NAD reported about 1% higher mortality than VS, and the 30-39 age group, where NAD reported slightly lower mortality (0.2-0.3%) than VS. Finally, VS included 32 cases under the “miscellaneous” category that did not appear in NAD; however, given the small number relative to total mortality, this discrepancy does not materially affect the overall equivalence between the 2 sources.

Table 3 summarizes the correlation analysis between VS and NAD across geographic levels and age groupings. The analysis was performed using 3 approaches: total deaths, life cycle stages (0-19, 20-64, 65-74, 75-84, ≥85), and 5-year intervals (0, 1-4, 5-9, …, 80-84, ≥85). Each correlation is further stratified by sex (total, male, and female), with the VS miscellaneous category excluded. The results show exceptionally high correlations in all categories, with coefficients consistently at or above 0.9999. At the national level, both male and female populations display nearly perfect correlations (1.0000) across all age groupings, with only a slight decrease in females for the life course grouping (0.9999).

At the provincial level, correlations remain extremely high (1.0000) across all categories, indicating strong consistency between VS and NAD regardless of sex or age grouping. The county-level analysis, while still demonstrating very high correlations (≥0.9996), shows slightly lower coefficients compared to national and provincial levels. This small decrease at the county-level likely reflects increased variability in smaller geographic units. The lowest observed correlation (0.9996) occurs in male county-level data for both life course and 5-year age groupings.

**Figure 1.**
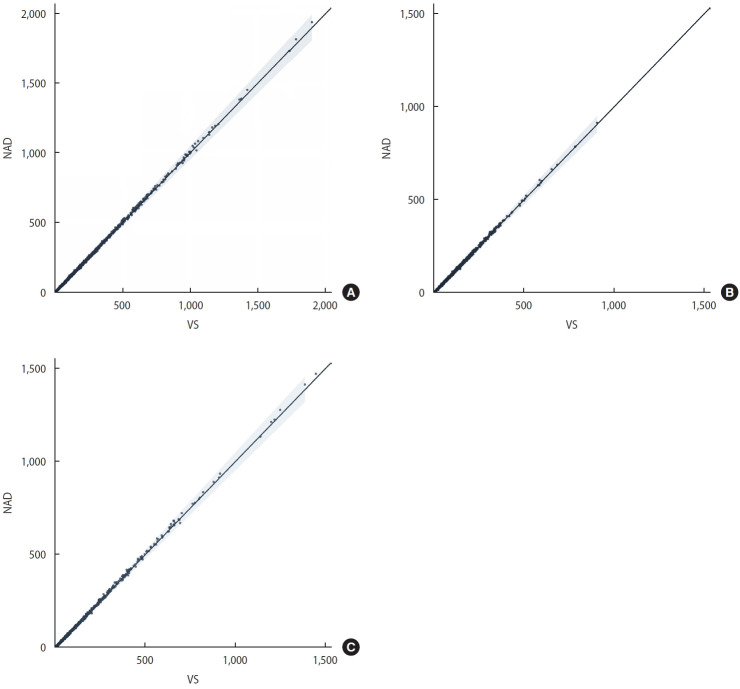
County-level age-specific mortality: correlation between vital statistics (VS) from Statistics Korea and National Administrative Data (NAD) from Ministry of Interior and Safety, 2023. (A) Total population, (B) male population, (C) female population.

Figure 1 displays scatter plots comparing mortality counts between VS and NAD at the county-level, with separate panels for the total population (A), male population (B), and female population (C). Each point represents an age-specific, sex-specific, and county-specific observation, with the solid black line indicating perfect agreement between the 2 sources. The shaded area marks a ±5% margin around the perfect agreement line.

The plots demonstrate very strong correlations between VS and NAD across all 3 population groups. Most points cluster very closely to the diagonal line, indicating high consistency between the sources. The tight grouping along the diagonal reflects the high correlation coefficients reported in Table 3 (0.9998, 0.9996, and 0.9998 for total, male, and female populations, respectively).

The scatter pattern is consistent across all panels, suggesting similarly strong agreement between VS and NAD regardless of sex. The points exhibit minimal dispersion, even at higher mortality counts, indicating that consistency remains robust across different mortality levels. This visual evidence supports the numerical findings that NAD could reliably substitute for VS in county-level mortality statistics, irrespective of population size or sex distribution.

**Figure 2.**
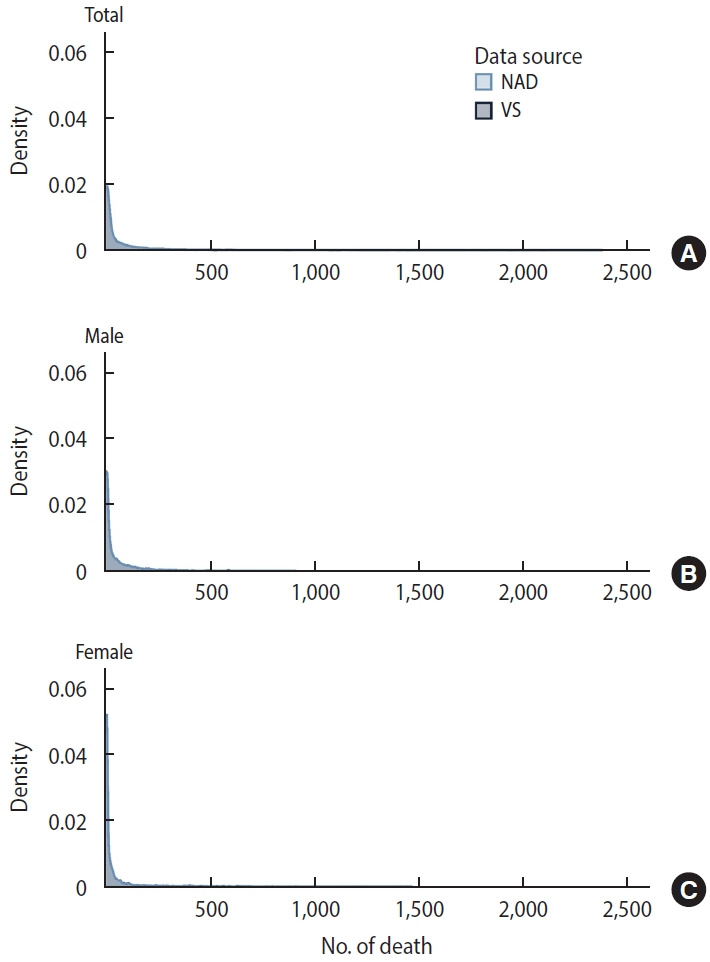
County-level age-specific mortality: distribution of vital statistics (VS) from Statistics Korea and National Administrative Data (NAD) from Ministry of Interior and Safety, 2023. (A) Total population, (B) male population, (C) female population.

Figure 2 presents density plots comparing the distribution of mortality counts between VS and NAD at the county-level, with separate panels for total population (A), male population (B), and female population (C). The plots show highly overlapping distributions, demonstrating a strong similarity in mortality patterns between the 2 sources. All panels display distinctly right-skewed distributions, with the highest density near zero deaths and long right tails extending toward higher mortality counts. This pattern aligns with the typical distribution of county-level mortality data, where most counties report low death counts and a few report high numbers. The nearly perfect overlap between VS (darker blue) and NAD (lighter blue) distributions, particularly in the peak densities near zero and throughout the right tails, further substantiates the strong correlation shown in Table 3.

## DISCUSSION

In this study, the comparison between VS and NAD revealed remarkably high consistency between the 2 datasets. At the national level, NAD reported only 0.4% more deaths than VS, with slight variations by sex (0.2% for male and 0.6% for female). Although notable differences were observed in early childhood mortality—16.8% lower in NAD for neonates and 14.8% higher for ages 1-4—as well as in certain months (5-9% differences), the overall agreement was strong, as demonstrated by very high correlation coefficients (≥0.9999) across all stratifications, especially at the national and provincial levels. Both scatter plots and density plots visually confirmed this strong agreement, showing tight clustering along the perfect agreement line and highly overlapping distributions. These findings suggest that NAD could serve as a reliable alternative to VS for real-time mortality surveillance.

Our focus on real-time mortality surveillance requires further attention to 2 key findings: variations in early childhood mortality and monthly fluctuations. In regard to early childhood mortality, we separated age 0 from ages 1-4, rather than using a combined 0-4 group, to reflect the relatively high mortality in the first year of life. Our analysis suggests that significant under-reporting or over-reporting in age-specific mortality can be mitigated when broader age groupings are used. While this issue may arise during periods of substantial year-to-year mortality change, the data demonstrates considerable stability when aggregated into 5-year or larger intervals. Therefore, the first key consideration when using NAD for surveillance is to avoid overly granular age groupings.

The second consideration involves monthly variations, which are especially relevant for surveillance applications. Analysis of 2023 data revealed alternating patterns of higher and lower mortality in NAD compared to VS: increases in February and March, decreases in April, increases in June, decreases in July and September, increases in October, and decreases in December. Given that these differences can be substantial, reaching up to 9.7% for male in February, monthly mortality analyses should incorporate sensitivity analyses with a 5-10% margin of error.

NAD offers a clear advantage over VS in terms of timeliness, with data available the following month compared to VS’s delay of up to 20 months. While NHIS data previously provided an advantage by offering sex-specific and age-specific mortality counts, this gap was closed in 2023 with the expanded availability of NAD data. However, a significant limitation of NAD is its lack of cause-of-death information, which remains a unique strength of VS and is essential for detailed epidemiological analysis and disease-specific mortality surveillance. This limitation means that NAD can track overall mortality patterns but cannot identify which causes are contributing to changes. Although NHIS data theoretically offers additional benefits—including consistent numerator-denominator identification for mortality rates and access to a variety of health indicators—these advantages have not led to practical applications [10], as evidenced by the absence of successful real-time mortality tracking studies using NHIS data. Moreover, the time required for data requests and acquisition limits these benefits to a small group of privileged researchers with early access to unpublished datasets.

Nevertheless, this study has important limitations. Since MOIS only began providing sex-specific and age-specific mortality data in 2023, long-term equivalence between NAD and VS requires further validation. While our analysis shows strong agreement for 2023, continued monitoring is necessary to determine whether these patterns persist in future years. For instance, a follow-up study comparing NAD and VS for 2023-2027 would be valuable. In addition, conducting various types of mortality analyses using NAD data would further clarify its characteristics and potential.

In this study, county-level mortality tracking using NAD from MOIS was validated to be nearly identical to and highly correlated with county-level data from VS of Statistics Korea. These results demonstrate that NAD can be effectively used for real-time mortality surveillance in Korea. A data source that is relatively simple but widely accessible may be more valuable than one that is comprehensive but restricted, particularly for real-time tracking during public health emergencies such as the COVID-19 pandemic. With sex-specific and age-specific data now available, NAD can serve as a crucial infrastructure for mortality surveillance in Korea.

## Figures and Tables

**Figure f3-epih-47-e2025042:**
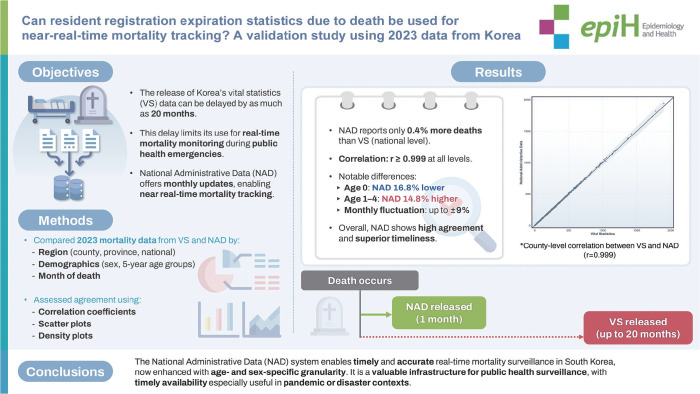


**Table 1.** Comparison of mortality counts between VS and NAD at national and provincial levels, 2023

Variables	Total		Male		Female	
	VS	NAD	VS	NAD	VS	NAD
National	352,511 (100)	353,920 (100.4)	188,921 (100)	189,341 (100.2)	163,590 (100)	164,579 (100.6)
Provincial						
Seoul	51,446 (100)	51,820 (100.7)	28,900 (100)	29,078 (100.6)	22,546 (100)	22,742 (100.9)
Busan	26,303 (100)	26,352 (100.2)	14,391 (100)	14,395 (100.0)	11,912 (100)	11,957 (100.4)
Daegu	16,556 (100)	16,272 (98.3)	8,844 (100)	8,695 (98.3)	7,712 (100)	7,577 (98.2)
Incheon	18,242 (100)	18,278 (100.2)	9,946 (100)	9,967 (100.2)	8,296 (100)	8,311 (100.2)
Gwangju	8,866 (100)	8,902 (100.4)	4,691 (100)	4,702 (100.2)	4,175 (100)	4,200 (100.6)
Daejeon	8,677 (100)	8,755 (100.9)	4,565 (100)	4,614 (101.1)	4,112 (100)	4,141 (100.7)
Ulsan	6,076 (100)	6,094 (100.3)	3,324 (100)	3,331 (100.2)	2,752 (100)	2,763 (100.4)
Sejong	1,595 (100)	1,595 (100.0)	828 (100)	836 (101.0)	767 (100)	759 (99.0)
Gyeonggi	74,949 (100)	75,373 (100.6)	40,580 (100)	40,661 (100.2)	34,369 (100)	34,712 (101.0)
Gangwon	14,224 (100)	14,231 (100.0)	7,684 (100)	7,672 (99.8)	6,540 (100)	6,559 (100.3)
Chungbuk	13,463 (100)	13,539 (100.6)	7,086 (100)	7,125 (100.6)	6,377 (100)	6,414 (100.6)
Chungnam	18,781 (100)	18,895 (100.6)	9,915 (100)	9,963 (100.5)	8,866 (100)	8,932 (100.7)
Jeonbuk	17,201 (100)	17,269 (100.4)	8,836 (100)	8,865 (100.3)	8,365 (100)	8,404 (100.5)
Jeonnam	19,786 (100)	19,837 (100.3)	10,015 (100)	9,990 (99.8)	9,771 (100)	9,847 (100.8)
Gyeongbuk	25,283 (100)	25,546 (101.0)	13,066 (100)	13,166 (100.8)	12,217 (100)	12,380 (101.3)
Gyeongnam	26,386 (100)	26,462 (100.3)	13,712 (100)	13,735 (100.2)	12,674 (100)	12,727 (100.4)
Jeju	4,677 (100)	4,700 (100.5)	2,538 (100)	2,546 (100.3)	2,139 (100)	2,154 (100.7)
Monthly						
January	32,658 (100)	32,749 (100.3)	17,409 (100)	17,185 (98.7)	15,249 (100)	15,564 (102.1)
February	27,350 (100)	29,787 (108.9)	14,575 (100)	15,984 (109.7)	12,775 (100)	13,803 (108.0)
March	28,944 (100)	30,324 (104.8)	15,506 (100)	16,172 (104.3)	13,438 (100)	14,152 (105.3)
April	27,539 (100)	25,593 (92.9)	14,934 (100)	13,781 (92.3)	12,605 (100)	11,812 (93.7)
May	28,876 (100)	29,458 (102.0)	15,558 (100)	15,965 (102.6)	13,318 (100)	13,493 (101.3)
June	26,794 (100)	28,867 (107.7)	14,450 (100)	15,506 (107.3)	12,344 (100)	13,361 (108.2)
July	28,141 (100)	26,538 (94.3)	15,167 (100)	14,335 (94.5)	12,974 (100)	12,203 (94.1)
August	30,523 (100)	30,802 (100.9)	16,354 (100)	16,536 (101.1)	14,169 (100)	14,266 (100.7)
September	28,293 (100)	26,257 (92.8)	15,357 (100)	14,122 (92.0)	12,936 (100)	12,135 (93.8)
October	30,793 (100)	32,510 (105.6)	16,521 (100)	17,471 (105.8)	14,272 (100)	15,039 (105.4)
November	30,364 (100)	30,561 (100.6)	16,123 (100)	16,298 (101.1)	14,241 (100)	14,263 (100.2)
December	32,236 (100)	30,474 (94.5)	16,967 (100)	15,986 (94.2)	15,269 (100)	14,488 (94.9)

Values are presented as mortality counts; Parentheses show the ratio of NAD to VS, with VS set to 100. VS, vital statistics; NAD, National Administrative Data.

**Table 2.** Age- and sex-specific mortality distribution: comparison between VS and NAD, 2023

Variables	Total		Male		Female	
	VS	NAD	VS	NAD	VS	NAD
Total	352,511 (100)	353,920 (100.4)	188,921 (100)	189,341 (100.2)	163,590 (100)	164,579 (100.6)
0	564 (100)	469 (83.2)	320 (100)	254 (79.4)	244 (100)	215 (88.1)
1-4	155 (100)	178 (114.8)	81 (100)	89 (109.9)	74 (100)	89 (120.3)
5-9	148 (100)	157 (106.1)	82 (100)	87 (106.1)	66 (100)	70 (106.1)
10-14	245 (100)	250 (102.0)	125 (100)	126 (100.8)	120 (100)	124 (103.3)
15-19	558 (100)	577 (103.4)	322 (100)	331 (102.8)	236 (100)	246 (104.2)
20-24	1,039 (100)	1,053 (101.3)	659 (100)	670 (101.7)	380 (100)	383 (100.8)
25-29	1,612 (100)	1,652 (102.5)	1,074 (100)	1,095 (102.0)	538 (100)	557 (103.5)
30-34	1,896 (100)	1,893 (99.8)	1,213 (100)	1,207 (99.5)	683 (100)	686 (100.4)
35-39	2,425 (100)	2,417 (99.7)	1,551 (100)	1,541 (99.4)	874 (100)	876 (100.2)
40-44	4,410 (100)	4,422 (100.3)	2,812 (100)	2,816 (100.1)	1,598 (100)	1,606 (100.5)
45-49	6,299 (100)	6,352 (100.8)	4,137 (100)	4,153 (100.4)	2,162 (100)	2,199 (101.7)
50-54	10,760 (100)	10,839 (100.7)	7,504 (100)	7,541 (100.5)	3,256 (100)	3,298 (101.3)
55-59	14,489 (100)	14,568 (100.5)	10,509 (100)	10,552 (100.4)	3,980 (100)	4,016 (100.9)
60-64	21,730 (100)	21,749 (100.1)	15,794 (100)	15,824 (100.2)	5,936 (100)	5,925 (99.8)
65-69	25,706 (100)	25,626 (99.7)	18,495 (100)	18,436 (99.7)	7,211 (100)	7,190 (99.7)
70-74	30,188 (100)	30,222 (100.1)	20,599 (100)	20,623 (100.1)	9,589 (100)	9,599 (100.1)
75-79	39,898 (100)	39,889 (100)	24,876 (100)	24,852 (99.9)	15,022 (100)	15,037 (100.1)
80-84	66,302 (100)	66,320 (100)	35,504 (100)	35,482 (99.9)	30,798 (100)	30,838 (100.1)
≥85	124,055 (100)	125,287 (101.0)	43,254 (100)	43,662 (100.9)	80,801 (100)	81,625 (101.0)
Miscellaneous	32 (100)	-	10 (100)	-	22 (100)	-

Values are presented as mortality counts; Parentheses show the ratio of NAD to VS, with VS set to 100. VS, vital statistics; NAD, National Administrative Data.

**Table 3.** Correlation analysis of regional age- and sex-specific mortality between VS and NAD, 2023

Region/Age group	Total	Life cycle stages (0-19, 20-64, 65-74, 75-84, ≥85)	5-year intervals (0, 1-4, 5-9, …, 80-84, ≥85)
Total			
National	-	1.0000	1.0000
Provincial	1.0000	0.9999	1.0000
County	0.9999	0.9998	0.9998
Male			
National	-	1.0000	1.0000
Provincial	1.0000	1.0000	1.0000
County	0.9999	0.9996	0.9996
Female			
National	-	0.9999	1.0000
Provincial	1.0000	1.0000	1.0000
County	0.9998	0.9997	0.9998

VS, vital statistics; NAD, National Administrative Data.
